# Cancer Epidemiology in the Northeastern United States (2013–2017)

**DOI:** 10.1158/2767-9764.CRC-23-0152

**Published:** 2023-08-14

**Authors:** Judy R. Rees, Julie E. Weiss, Christine M. Gunn, Heather A. Carlos, Nathalie C. Dragnev, Emma Y. Supattapone, Anna N.A. Tosteson, Sally A. Kraft, Linda T. Vahdat, Janet L. Peacock

**Affiliations:** 1 Department of Epidemiology, Geisel School of Medicine at Dartmouth, Hanover, New Hampshire.; 2 Dartmouth Cancer Center, Lebanon, New Hampshire.; 3 The Dartmouth Institute for Health Policy and Clinical Practice, Geisel School of Medicine at Dartmouth, Hanover, New Hampshire.; 4 Wake Forest University School of Medicine, Winston-Salem, North Carolina.; 5 Dartmouth College, Hanover, New Hampshire.; 6 Dartmouth Health, Lebanon, New Hampshire.

## Abstract

We tested the hypotheses that adult cancer incidence and mortality in the Northeast region and in Northern New England (NNE) were different than the rest of the United States, and described other related cancer metrics and risk factor prevalence. Using national, publicly available cancer registry data, we compared cancer incidence and mortality in the Northeast region with the United States and NNE with the United States overall and by race/ethnicity, using age-standardized cancer incidence and rate ratios (RR). Compared with the United States, age-adjusted cancer incidence in adults of all races combined was higher in the Northeast (RR, 1.07; 95% confidence interval [CI] 1.07–1.08) and in NNE (RR 1.06; CI 1.05–1.07). However compared with the United States, mortality was lower in the Northeast (RR, 0.98; CI 0.98–0.98) but higher in NNE (RR, 1.05; CI 1.03–1.06). Mortality in NNE was higher than the United States for cancers of the brain (RR, 1.16; CI 1.07–1.26), uterus (RR, 1.32; CI 1.14–1.52), esophagus (RR, 1.36; CI 1.26–1.47), lung (RR, 1.12; CI 1.09–1.15), bladder (RR, 1.23; CI 1.14–1.33), and melanoma (RR, 1.13; CI 1.01–1.27). Significantly higher overall cancer incidence was seen in the Northeast than the United States in all race/ethnicity subgroups except Native American/Alaska Natives (RR, 0.68; CI 0.64–0.72).

In conclusion, NNE has higher cancer incidence and mortality than the United States, a pattern that contrasts with the Northeast region, which has lower cancer mortality overall than the United States despite higher incidence.

**Significance:**

These findings highlight the need to identify the causes of higher cancer incidence in the Northeast and the excess cancer mortality in NNE.

## Introduction

Rural regions experience specific challenges in cancer prevention, incidence, mortality, and survival. The Centers for Disease Control and Prevention report that nonmetropolitan rural counties have higher cancer mortality than urban areas, and cancer mortality disparities in rural versus urban counties are increasing (1). Differences between urban and rural regions are seen for cancer risk factors such as smoking and obesity, and rural areas have poorer access to resources to address them (2–4). A variety of environmental and other cancer risk factors that may be associated with excess cancer risk in Northern New England (NNE) include home radon levels (5), sun exposure (6), smoking (7), and contamination of drinking water by arsenic (8, 9), nitrates (10), and by perfluoralkyl substances (11). These issues are compounded by differences in health-related beliefs and behaviors, and access to and use of preventive care (12–15). As such, smoking-related cancers and screen-preventable cancers have higher incidence in rural counties and these disparities are also increasing over time (1). In the Northeast, travel distances for preventive care and cancer treatment can be challenging, particularly during winter months, and rural/urban differences in cancer treatment have been documented (16–19).

The Northeast region of Connecticut, Maine (ME), Massachusetts, New Hampshire (NH), New Jersey, New York, Pennsylvania, Rhode Island, and Vermont (VT), includes NNE, comprising ME, NH, and VT, three small states that share many common geographic features and predominantly White populations (20–24). Rural residents comprise 52%, 40%, and 71% of the populations of ME, NH, and VT, respectively, compared with 25% of the United States as a whole. We used epidemiologic data to test whether cancer incidence and mortality in NNE and the Northeast are different than the rest of the United States overall and by race/ethnicity. We also present summary descriptive data on 5-year survival, annual percent change in incidence and mortality, cancer risk factors, and cancer registry data quality that may influence the trends observed.

## Materials and Methods

### Data Sources

**FIGURE 1:**
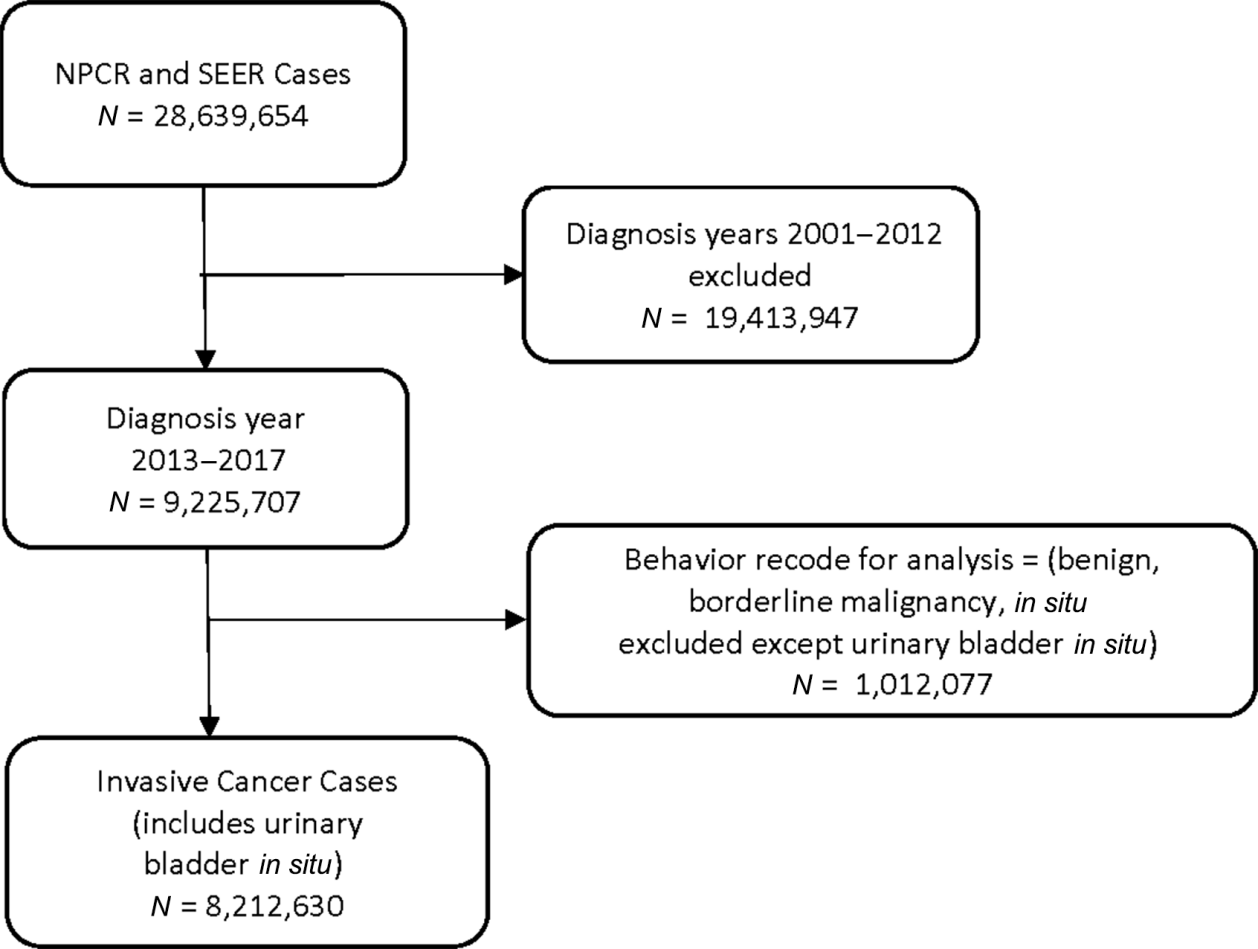
Derivation of cancer cases.

In analyses overall and by race/ethnicity, we used data from the National Program of Cancer Registries (NPRC) and Surveillance, Epidemiology, and End Results (SEER) analytic files, “NPCR and SEER Incidence—U.S. Cancer Statistics Public Use Database, 2019 (2001–2017)” (25) and “Mortality—All COD, Aggregated with State, Total U.S. (1990–2017)” (26) to compare cancer incidence and mortality in the United States during 2013–2017 with the Northeast region and with the three NNE states of ME, NH, and VT during 2013–2017 (Fig. 1). Analyses were restricted to tumor behavior coded as “malignant, only malignant in International Classification of Diseases for Oncology, 3rd edition (ICD-O-3; ref. 27) and malignant 2010+ cases” (27). All eligible primary cancers for every patient were included. Comparison of the three NNE states with the United States was conducted using age-standardized cancer incidence and rate ratios (RR). Analyses were conducted using SEER*Stat version 8.3.9 (28), which automatically suppressed cell sizes of <16 for rate stability (25).

Race/ethnicity was defined by combining two variables: (i) race recode [White, Black, American Indian/Alaska Native (AIAN), Asian/Pacific Islander (API), Unknown] and (ii) Origin Recode NAACCR Hispanic Identification Algorithm (ref. 29; Hispanic, Non-Hispanic) to create a variable that defined Non-Hispanic White, Non-Hispanic Black, Non-Hispanic AIAN, Non-Hispanic API, and Hispanic (all races; ref. 30). Overall case counts included individuals with unknown race or ethnicity.

Descriptive data that are presented include relative survival ratios (RSR) and their 95% confidence intervals (CI) from “Cancer in North America, Volume 4: Cancer Survival in the United States and Canada 2012–2018” (31). Annual percent changes in incidence and mortality were taken from publicly available aggregate data (32). Aggregated data on state characteristics, demographics, health care factors, cancer screening, and behavioral risk factors are presented for the United States, ME, NH, and VT using data from publicly available sources (5, 20, 23, 24, 33–58). National Priorities List (NPL) sites have been defined by the U.S. Environmental Protection Agency as areas of the United States and its territories that merit further investigation due to pollution or contamination by hazardous substances (59). Metrics for NPL sites were calculated by summing the numbers of NPL sites (past, present, and proposed) for each state (34–36), and calculating the totals relative to the state population (37), or the state land area in square miles (20). For U.S. states, the median value and range for these metrics are reported.

Case completeness (an estimate of the proportion of cases in the community that are recorded in the registry) for all three states met the 95% standard of the NPRC in all 5 years (Supplementary Table S1). Using a nationally recognized formula based on the ratio of incidence to mortality (60), the range of estimated case completeness at 24 months during the period 2013–2017 tended to be lower for VT (101.2%–106.9%) than ME (105.7%–111.1%) and NH (104.3%–111.9%; ref. 61).

### Statistical Analysis

Statistical analysis followed methods we have described previously (62). In brief, cancer incidence, mortality, and late-stage cancer incidence were directly age-standardized to the 2000 U.S. population and presented as incidence per 100,000. To compare each state with the United States, we calculated the ratio of the appropriate pair of age-standardized rates. Tiwari method was used to calculate SEs and CI (63). Bonferroni correction was used to adjust for multiple testing of differences in incidence/mortality rates between the United States and the three NNE states (the race/ethnicity subanalysis included U.S. regions). Nominal 95% CI were calculated using the same correction factor, that is, giving “99%” CIs. Footnotes below each table or figure indicate any corrections that were applied. RRs and their 99% CIs are presented in forest plots for the primary analyses (incidence and mortality) so that statistical significance at alpha = 0.05, after adjustment for multiple testing, is shown by a CI that does not cross 1.0. Forest plots could not be created when data were suppressed in SEER*Stat; this was the case for cell counts <16 for incidence and <10 for mortality. Survival analyses could not be conducted in a comparable way to analyses described here due to state data limitations, and so we simply present the descriptive data by state.

### Data Availability Statement

The data are available from the NPRC and SEER analytic files, “NPCR and SEER Incidence—U.S. Cancer Statistics Public Use Database, 2019 (2001–2017)” (25) and “Mortality—All COD, Aggregated with State, Total U.S. (1990–2017)” (26).

## Results

### Incidence

**FIGURE 2:**
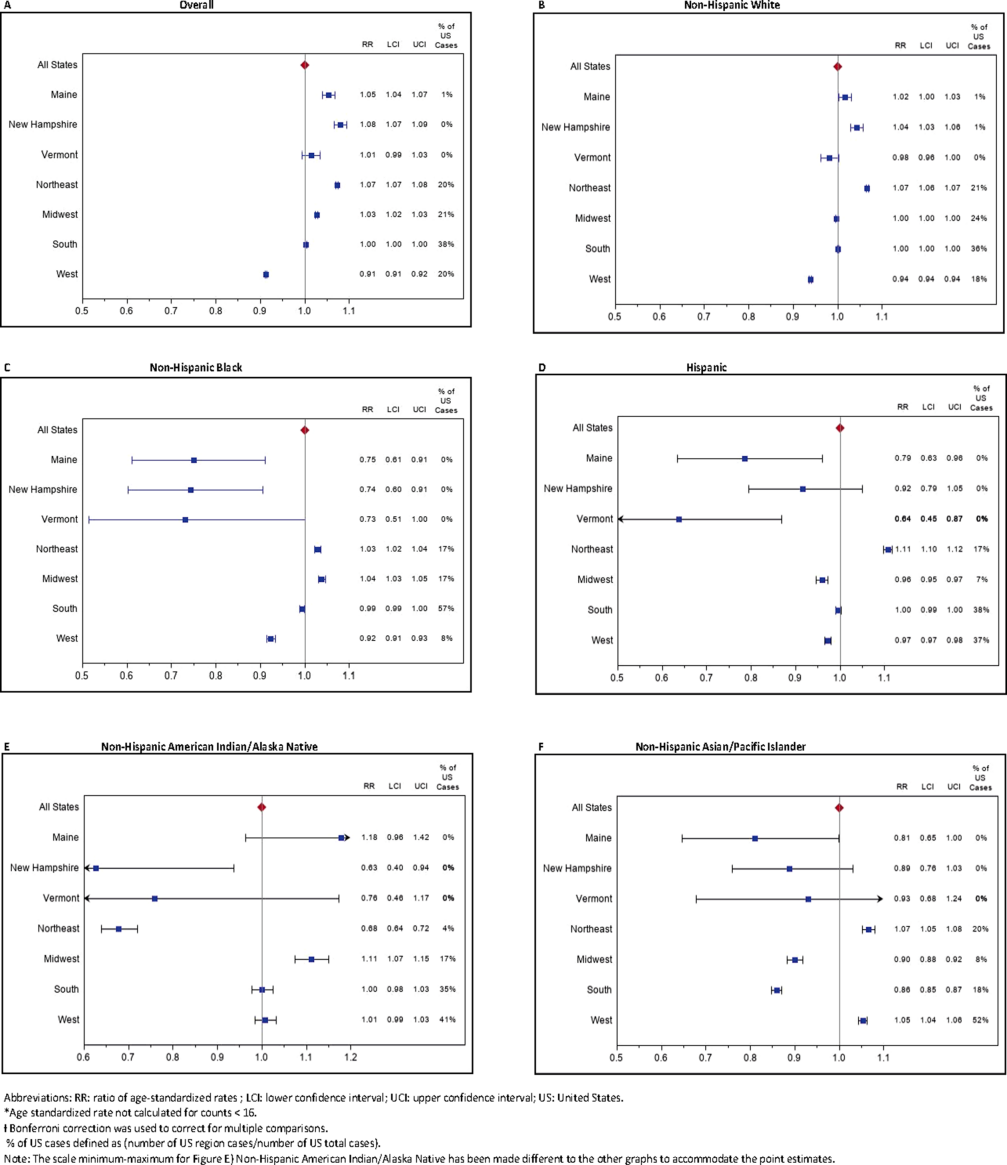
Ratios of age-standardized* cancer incidence rates (RR) and 99% CIs† for ME, NH, VT, and U.S. regions compared with United States, by race/ethnicity 2013–2017.

**FIGURE 3:**
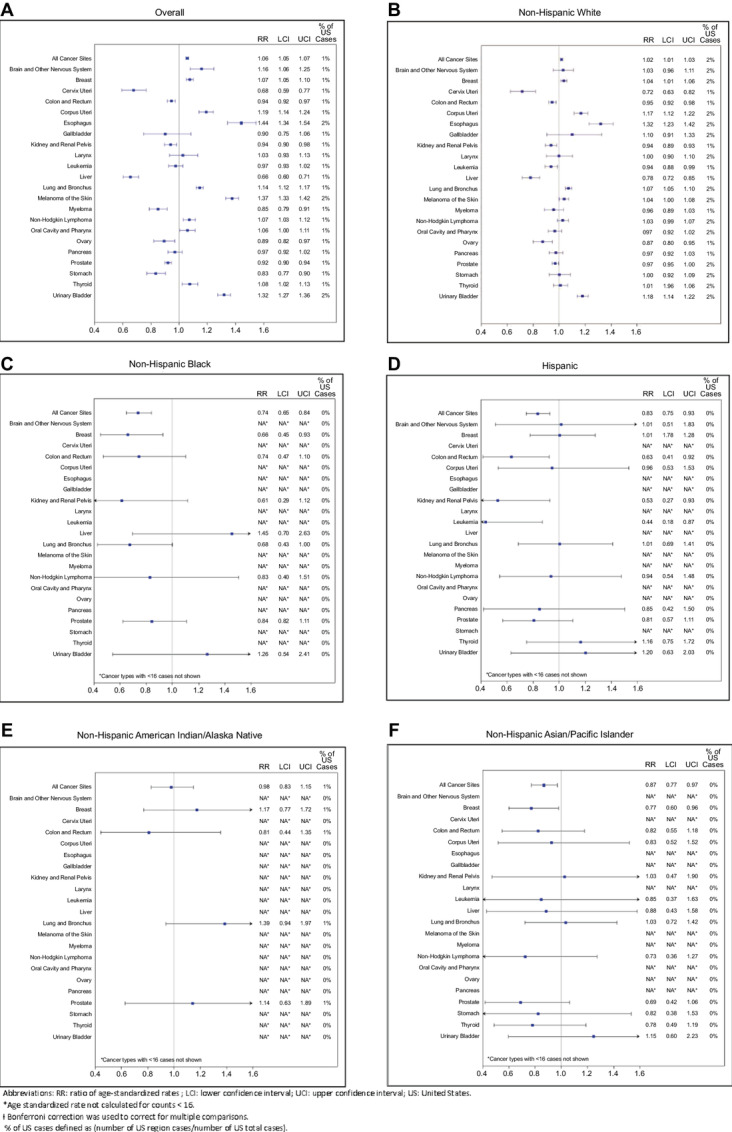
Leading cancer sites ratio of age-standardized* incidence rates (RR) and 99% CIs† for combined ME, NH, VT versus the United States, by race/ethnicity 2013–2017.

Age-adjusted cancer incidence in adults showed substantial variation by region (Supplementary Fig. S1), and was highest over all races in the Northeast compared with the United States (RR, 1.07; CI 95% CI, 1.07–1.08; Fig. 2; Supplementary Table S2). Comparing NNE with the United States, the age-adjusted incidence was also higher overall (RR, 1.06; CI 1.05–1.07) and for five cancer types among the non-Hispanic White population in NNE (bladder, female breast, esophagus, lung/bronchus, and uterus; Fig. 3; Supplementary Table S3). Six were significantly less common: cervix, colon and rectum, kidney, leukemia, liver, and ovary. Among non-Hispanic Black and Asian/Pacific Islander populations, cancer incidence overall and female breast cancer in NNE were significantly lower than the rest of the United States overall. Among Hispanic individuals (all races), cancer incidence in NNE was significantly lower than the rest of the United States overall, and specifically for colorectal and kidney cancers and leukemia.

Among all races combined, some variations were seen within NNE (Supplementary Table S2); where there were differences, they tended to reflect lower incidence in VT than one or both of the other two states. In particular, NH's female breast and prostate cancer incidences were significantly higher than both other states. Melanoma incidence was very different in the three states with large interstate differences (VT>NH>ME).

At the regional level, incidence was higher in the Northeast among all minority populations except non-Hispanic American Indian/Alaska Native populations, whose incidence was significantly lower in the Northeast than the rest of the United States (Fig. 2; Supplementary Table S4). Despite higher incidence in the non-Hispanic Black population in the Northeast than in the United States, non-Hispanic Black individuals in NH and ME had significantly lower cancer incidence, with a similar but nonsignificant pattern in VT. Similarly, lower incidence was seen in the Hispanic population and possibly also among non-Hispanic Asian/Pacific Islanders in the three NNE states, despite a similar pattern of higher incidence in these minority groups in the whole Northeast region.

### Late-stage Incidence

Incidence of late-stage cancers largely mirrored that of all invasive cancers, with significantly elevated rates in ME and NH but not VT when compared with the United States (Table 1). Incidence of lung/bronchus cancer diagnosed overall (Supplementary Table S2) or at late stage was significantly higher in all three NNE states than in the United States. ME had the highest incidence of late-stage cancer among the three states compared with the United States. Incidence of late-stage prostate cancers was significantly higher in each of the three states than in the United States, despite differences in overall prostate cancer incidence in ME and VT (significantly lower than United States) and NH (significantly higher than United States; Fig. 3). For cancer of the uterus, although overall incidence in each of the three states significantly exceeded that in the United States (Supplementary Table S2), diagnosis at late stage was not significantly higher in any of the three states when compared with the United States (Table 1).

**TABLE 1:** Age-adjusted incidence of late-stage cancers^a^^,^^b^, all Races, for ME, NH, VT, and the United States, 2013–17

Cancers diagnosed at regional or distant stage	All states (Population: 1,603,405,284)		Maine (Population: 6,653,873)		New Hampshire (Population: 6,688,065)		Vermont (Population: 3,124,796)	
Rate (95% CI)	Count	Rate (95% CI)	Count	Rate (95% CI)	Count	Rate (95% CI)	Count
All Invasive	203.9 (203.7–204.1)	3,794,100	218.8^c^ (215.7–221.9)	20,349	210.9^c^ (207.7–214.1)	18,100	201.3 (196.8–205.8)	8,308
Brain and other nervous system	1.0 (0.9–1.0)	16,873	1.1 (0.8–1.4)	81	1.3^c^ (1.0–1.6)	100	1.4 (1.0–1.9)	49
Breast (Female)	42.0 (41.9–42.1)	396,860	39.5 (37.6–41.5)	1,783	43.8 (41.7–45.9)	1,869	36.2^c^ (33.5–39.1)	726
Cervix uteri	3.6 (3.6–3.7)	31,871	2.5^c^ (2.0–3.1)	103	2.0^c^ (1.5–2.5)	81	1.5^c^ (0.9–2.2)	26
Colon and rectum	21.6 (21.6–21.7)	399,542	20.0^c^ (19.1–21.0)	1,845	20.5 (19.5–21.5)	1,740	20.1 (18.7–21.6)	819
Corpus uteri	7.0 (6.9–7.0)	71,609	6.0 (5.3–6.8)	309	6.7 (5.9–7.5)	319	7.4 (6.3–8.7)	167
Esophagus	3.1 (3.1–3.1)	59,417	5.0^c^ (4.6–5.5)	489	4.7 (4.2–5.1)	420	4.0^c^ (3.4–4.7)	178
Gallbladder	0.9 (0.9–0.9)	16,752	0.6^c^ (0.5–0.8)	60	0.9 (0.7–1.1)	73	0.9 (0.6–1.2)	38
Kidney and renal pelvis	4.8 (4.8–4.9)	90,917	4.9 (4.4–5.3)	467	4.7 (4.2–5.2)	410	4.6 (4.0–5.3)	197
Larynx	1.4 (1.3–1.4)	26,719	1.4 (1.2–1.7)	140	1.1 (0.9–1.4)	105	1.3 (1.0–1.7)	55
Leukemia	13.8 (13.8–13.9)	248,955	14.3 (13.4–15.1)	1,271	13.5 (12.7–14.4)	1,088	11.7^c^ (10.6–12.9)	460
Liver	2.8 (2.7–2.8)	54,752	1.9^c^ (1.6–2.2)	194	1.9^c^ (1.6–2.2)	174	2.0^c^ (1.6–2.5)	84
Lung and bronchus	40.6 (40.5–40.7)	775,215	51.8^c^ (50.3–53.3)	5,028	45.0^c^ (43.6–46.5)	4,013	44.3^c^ (42.3–46.5)	1,899
Melanoma of the skin	3.1 (3.0–3.1)	55,562	3.2 (2.9–3.7)	285	3.9^c^ (3.5–4.4)	317	3.6 (3.0–4.3)	141
Myeloma	6.4 (6.4–6.5)	121,316	5.6^c^ (5.1–6.1)	541	5.6^c^ (5.1–6.1)	490	5.1^c^ (4.4–5.9)	222
Non–Hodgkin lymphoma	12.1 (12.1–12.2)	223,113	14.7^c^ (13.9–15.5)	1,353	13.7^c^ (12.9–14.6)	1,161	14.0^c^ (12.9–15.3)	571
Oral cavity and pharynx	7.3 (7.3–7.4)	141,164	8.4^c^ (7.8–9.0)	791	7.5 (6.9–8.1)	686	7.9 (7.0–8.8)	346
Ovary	8.1 (8.0–8.1)	79,701	7.1 (6.3–8.0)	341	7.7 (6.9–8.6)	362	7.5 (6.4–8.9)	163
Pancreas	9.9 (9.8–9.9)	187,557	9.9 (9.2–10.5)	965	9.9 (9.2–10.6)	872	9.0 (8.1–10.0)	384
Prostate	21.0 (20.9–21.1)	193,682	22.9^c^ (21.5–24.4)	1,098	24.0^c^ (22.6–25.6)	1,060	24.3^c^ (22.2–26.6)	524
Stomach	3.8 (3.7–3.8)	70,168	3.1^c^ (2.7–3.5)	298	2.9^c^ (2.5–3.3)	255	3.4 (2.8–4.0)	138
Thyroid	4.4 (4.4–4.4)	73,352	4.9 (4.4–5.5)	355	4.5 (4.0–5.0)	321	4.6 (3.9–5.4)	153
Urinary bladder (includes *in situ*)	2.4 (2.3–2.4)	44,285	3.2^c^ (2.9–3.6)	311	3.2^c^ (2.8–3.6)	274	3.0 (2.5–3.6)	124

NOTE: Statistics not displayed because of <6 cases to preserve confidentiality.

^a^Rates are per 100,000, age-adjusted to the 2000 U.S. Std Population (19 age groups – Census P25–1130); confidence intervals using Tiwari method (63) are 95%.

^b^Late stage is defined as cases determined to be regional or distant. Coding is generally based on SEER summary stage but may include other staging variables if necessary.

^c^The rate ratio indicates that the rate is significantly different than the rate for all states after adjusting for multiple testing (*P* < 0.01).

Data: NPCR and SEER Incidence – U.S. Cancer Statistics Public Use Database, 2019 submission (2001–2017) (25).

Software: Surveillance Research Program, National Cancer Institute SEER*Stat software (www.seer.cancer.gov/seerstat) version 8.3.9 (28).

### Annual Percent Change in Incidence

Considering all cancers, races, and ethnicities combined, the burden from cancer incidence in the United States decreased significantly between 2014 and 2018 by about 1% per year (Supplementary Fig. S2A). Incidence is increasing nationally for cancers of the uterus, pancreas, and female breast but the frequency of many other cancer subtypes is decreasing. Potentially notable differences in these trends include ME's and NH's statistically significant increases in cancers of the kidney and liver/bile duct which were not seen nationally. However, incidence rates in these states remain below those in the United States overall. ME and VT have seen significant increases in melanoma of the skin, and ME had a significant increase in cancer of the oral cavity and pharynx. Significant increases in cancer of the uterus were seen in the United States and in VT.

### Mortality

**FIGURE 4:**
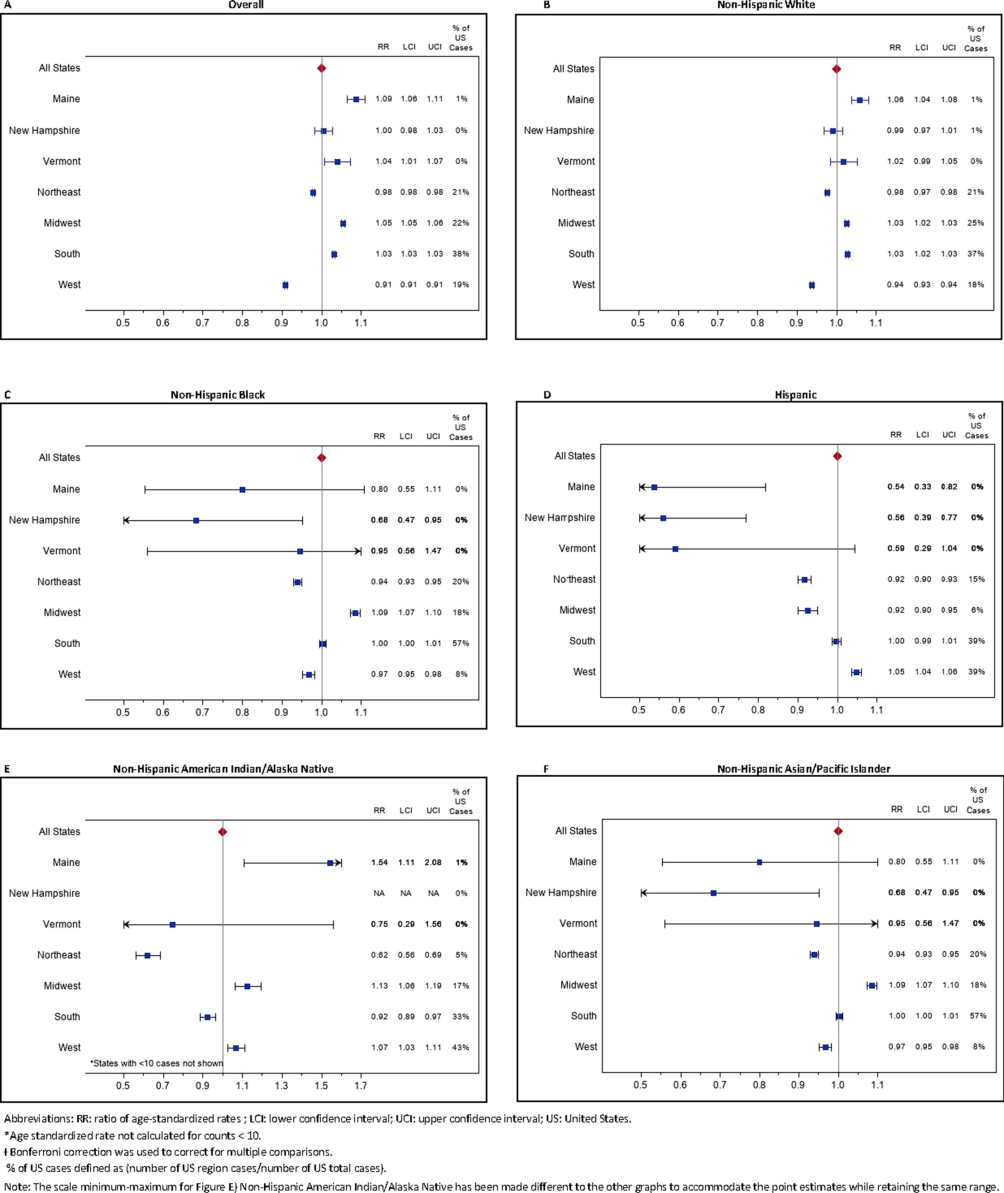
Ratios of age-standardized* cancer mortality rates (RR) and 99% CIs† for ME, NH, VT, and U.S. regions compared with United States, overall and among non-Hispanic White 2013–2017.

**FIGURE 5:**
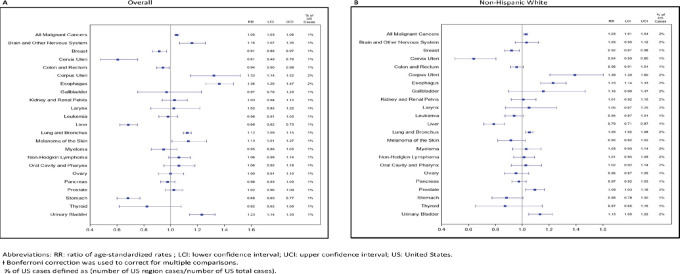
Leading cancer sites ratio of age-standardized mortality rates (RR) and 99% CIs† for combined ME, NH, VT versus the United States, overall and among non-Hispanic White 2013–2017.

Whereas the Northeast had the highest cancer incidence relative to the United States (Fig. 2), it had significantly lower cancer mortality overall when compared with the United States as a whole (Fig. 4). However, NNE had significantly higher incidence (Fig. 3) and mortality than the United States (Fig. 5; Supplementary Table S5). Furthermore, in ME and VT individually, cancer mortality was significantly higher overall than the United States or indeed the Northeast region (Fig. 4). A similar pattern was seen for non-Hispanic White only, although VT's higher mortality was not statistically significantly higher than the United States. For all race/ethnicity groups, mortality was lower in the Northeast with similar patterns among all three states although some CIs were wide due to small numbers, and ME's non-Hispanic American Indian/Alaska Native mortality stood out as significantly higher than their counterparts in the United States.

Because of the small numbers, we could not undertake meaningful analyses of cancer subtypes by race/ethnicity. However, in the tri-state NNE area analyzed together, restricted to non-Hispanic White individuals, significantly higher mortality was seen for cancers of the esophagus, lung/bronchus, prostate, urinary bladder, and uterus (Supplementary Table S3). Significantly lower mortality was seen for cancers of the breast, cervix, and liver. Several cancers showed patterns of mortality that matched the pattern of incidence, for example, for non-Hispanic White individuals, either both were higher (esophagus, lung/bronchus, bladder, uterus) or both lower (cervix, liver) than the United States. However, breast cancer had significantly higher incidence in NNE than the United States, but mortality was significantly lower. In contrast, prostate cancer incidence was lower in NNE than the United States, but mortality was significantly higher.

Better 5-year relative survival than the United States was seen in NH and VT (Table 2), but the available data were not examined by stage at diagnosis or race.

**TABLE 2:** Five-year age-standardized relative survival ratios for cancers diagnosed 2012–2018, follow-up through 2019 by registry, all races, for ME, NH, VT with the United States

	All states		Maine		New Hampshire		Vermont	
Cancer sites	RSR (95% CI)	Count	RSR (95% CI)	Count	RSR (95% CI)	Count	RSR (95% CI)	Count
All invasive	64.9 (64.9–64.9)	9,974,194	65.6 (65.0–66.2)	56,613	68.6 (68.0–69.2)	53,656	67.1 (66.1–68.0)	24,400
Brain and other nervous system	30.5 (30.2–30.8)	128,401	30.7 (26.9–34.6)	781	33.5 (29.2–37.9)	714	28.6 (22.9–34.5)	324
Breast (female)	89.6 (89.5–89.7)	1,526,050	92.3 (90.6–93.7)	8,120	92.5 (90.8–94.0)	8,516	92.1 (89.7–93.9)	3,701
Cervix uteri	61.5 (61.0–62.0)	83,219	60.3 (54.0–66.0)	284	66.5 (58.6–73.2)^a^	246	70.0 (57.8–79.3)	96
Colon and rectum	63.4 (63.2–63.5)	881,080	66.9 (64.7–69.1)	4,460	663 (63.9–68.5)	4,148	59.8 (56.4–63.1)	1,933
Corpus uteri	77.9 (77.6–78.2)	351,351	82.8 (79.1–85.9)	2,265	80.7 (77.0–83.8)	2,087	83.1 (78.5–86.8)	1,105
Esophagus	20.8 (20.4–21.1)	109,130	22.6 (18.6–26.9)^a^	890	25.8 (21.6–30.2)	831	27.6 (21.7–33.9)^a^	347
Kidney and renal pelvis	73.5 (73.2–73.7)	391,774	73.1 (69.9–76.0)	2,102	77.1 (73.4–80.3)	1,849	72.2 (66.3–77.3)	836
Larynx	59.9 (59.3–60.4)	80,065	61.8 (55.2–67.6)	501	59.5 (51.9–66.4)	391	54.9 (41.5–66.5)	190
Leukemia	58.2 (57.9–58.4)	308,382	62.1 (58.8–65.2)	1,736	60.8 (57.3–64.1)	1,527	58.4 (52.6–63.7)	656
Liver	19.1 (18.9–19.4)	204,734	16.2 (12.2–20.8)	721	19.5 (15.5–23.8)	736	20.4 (14.9–26.5)^a^	323
Lung and bronchus	25.0 (24.8–25.1)	1,351,208	23.8 (22.4–25.3)	9,173	26.9 (25.3–28.7)	7,386	28.1 (25.7–30.6)	3,501
Melanoma of the skin	92.6 (92.4–92.7)	494,463	94.3 (92.4–95.7)	3,085	93.7 (91.7–95.2)	3,435	95.4 (92.4–97.3)	1,921
Myeloma	57.1 (56.7–57.4)	165,750	55.6 (50.2–60.6)	795	63.7 (58.3–68.6)	731	57.8 (49.2–65.5)	327
Non–Hodgkin lymphoma	71.1 (70.9–71.3)	441,540	74.1 (71.3–76.6)	2,574	76.0 (73.0–78.8)	2,409	75.2 (70.8–79.1)	1,118
Oral cavity and pharynx	62.2 (61.9–62.5)	280,643	64.2 (60.3–67.8)	1,711	65.4 (60.7–69.6)	1,434	63.2 (56.7–69.0)	697
Ovary	44.5 (44.1–44.9)	133,707	48.7 (43.3–53.9)	596	42.7 (37.5–47.8)	650	45.2 (37.2–52.9)	302
Pancreas	13.8 (13.6–14.0)	302,735	11.9 (9.4–14.8)	1,670	16.1 (13.3–19.0)	1,507	16.2 (12.1–20.7)	709
Prostate	94.5 (94.4–94.7)	1,275,778	93.0 (90.5–94.8)	6,241	93.2 (91.0–94.9)	7,083	91.2 (87.6–93.8)	2,755
Stomach	34.6 (34.3–35.0)	153,608	39.1 (34.2–43.9)	719	41.4 (35.7–47.1)*	639	33.1 (25.8–40.6)	296
Thyroid	96.2 (96.0–96.4)	305,834	97.0 (95.1–98.2)	1,580	97.2 (94.6–98.5)	1,594	92.6 (88.7–95.2)	671
Urinary bladder (includes *in situ*)	76.6 (76.4–76.8)	470,540	80.1 (77.6–82.3)	3,523	79.6 (77.0–82.0)	3,247	76.3 (72.4–79.7)	1,359

Abbreviations: CI, confidence interval; RSR, relative survival ratio.

^a^RSR and CI values are not age-standardized.

Data: https://www.naaccr.org/wp-content/uploads/2020/06/CINA.2013–2017.v4.survival.pdf (31).

### Annual Percent Change in Mortality

Considering all cancers, races, and ethnicities combined from 2014 to 2018, mortality in the United States decreased for most cancers (Supplementary Fig. S2B), including screen-amenable cancers (cervix, breast, prostate, colorectal, lung, melanoma) and those for which screening is not currently available (bladder, stomach, esophagus, non–Hodgkin lymphoma, leukemia, kidney, ovary). However, mortality from cancer of the uterus increased significantly in ME and NH and in the United States. Mortality due to cancer of the liver and bile duct increased significantly in NH and VT, but not the United States. A significant decrease in mortality from cancer of the oral cavity and pharynx was seen in NH, contrasting with a significant increase in mortality nationwide.

### Cancer Risk Factors

Publicly available data were identified for several important cancer incidence and mortality risk factors (Supplementary Table S6), for example, cigarette tax ranges from $1.78 in NH to $3.08 in VT (50); median household income ranges from $59,489 in ME to $77,923 in NH (42), and the number of physicians per 100,000 population ranges from 374.0 in ME to 517.8 in VT (47). Prevalence of ever smoking and/or heavy drinking is relatively high in NNE (47, 51). NNE colorectal cancer and lung cancer screening metrics appear to be good (5, 51), and human papilloma virus vaccination prevalence is also relatively high, but there is some variability in breast, cervical, and prostate cancer screening metrics (51). NNE women have low prevalence of hysterectomy (64) and low fertility rates (33). Despite high proportions of rural residence (from 39.6% in NH to 71.2% in VT, compared with 25.5% in the United States; ref. 23), there were substantially higher total numbers per 10,000 land miles of past, present, and proposed Superfund NPL sites (34–36) reflecting hazardous contamination in VT (14.6) and NH (23.5), compared with 5.2 nationally and in ME.

## Discussion

Cancer incidence in the United States is known to be highest in the Northeast (52), but this report also considers patterns of adult cancer in the predominantly white, rural, NNE area of ME, NH and VT. Overall, NNE has higher incidence than the rest of the country for 10 cancers (brain, female breast, uterus, esophagus, lung/bronchus, melanoma, non–Hodgkin lymphoma, oral cavity/pharynx, thyroid, and bladder), and significantly lower for eight (cervix, colon/rectum, kidney, liver, myeloma, ovary, prostate, stomach). When restricted to the majority non-Hispanic White population, fewer cancers stand out for high incidence: breast, uterus, esophagus, lung/bronchus, and bladder. Mortality overall was significantly higher in NNE for six cancers (brain, uterus, esophagus, lung/bronchus, bladder, and melanoma), and significantly lower for five (female breast, cervix, colorectum, liver, stomach). Among non-Hispanic Whites, mortality was significantly higher for cancers of the uterus, esophagus, lung, prostate, and bladder.

While NNE has higher cancer incidence than the United States overall and among non-Hispanic White populations, its non-Hispanic Black, Hispanic, and API populations appear to have lower overall cancer incidence than the same minorities in the United States. One possible explanation relates to the high population movement into NNE when compared with the United States as a whole (65); it is possible that minorities migrating into the NNE are healthier than those who do not migrate, leading to lower cancer risk in some population subgroups. A second possible explanation relates to differences in uptake of screening by race, ethnicity, and other sociodemographic factors (66, 67); lower prevalence of screening may reduce cancer incidence due to overdiagnosis (pseudodisease). Third, within a predominantly white area, more minorities may be misclassified as white in medical records because of poor data collection methods. In 2005, race/ethnicity data collection in NH hospitals was not generally based on self-identification but on assumptions made by clerical staff (68) which is likely prone to error. Fourth, there may be poorer local ascertainment of cancer outcomes in migrant populations if they are very mobile from year to year. There is evidence that health care facilities may still collect flawed, inconsistent race/ethnicity data (69–75), leading to misclassification of racial minority status in patients with cancer. However, the lower cancer incidence among northeastern Native Americans/Alaska Native populations argues against the misclassification hypothesis at least in this minority group, because state cancer registries routinely link with the Indian Health Service database to optimize ascertainment of cancers among the 2.6 million Native Americans/Alaska Natives within the United States (76).

Many different cancer risk factors in this rural, tri-state environment may contribute to some of these patterns of incidence and mortality, including smoking (77), arsenic in well water (78), radon in air (79), and pollution; both NH and VT have more than three times the number of NPL sites (past, current, and proposed) per square mile than the United States average (34–36), as well as specific issues with contaminants such as perfluoralkyl substances (11, 80). Substantial proportions of the NNE population are exposed to arsenic through well water (78), as a large proportion of residents in NNE use private wells as their drinking water supply, and the high prevalence of arsenic and nitrates in the tri-state area is thought to contribute to high bladder cancer rates (8, 10, 81, 82). In addition to the burden of lung cancer due to smoking—with sex-specific differences that may drive some of the trends we report (77)—it was estimated in 2003 that unmitigated radon in homes throughout the United States accounted for more than 21,100, or 13.4% of lung cancers each year (83). An estimated 1 in 15 (7%) homes in the United States exceed the EPA action level of 4pCi/L for indoor air, but the figures are much higher in VT (22%), NH (35%), and ME (37%; refs. 5, 84). The risk of lung cancer in smokers exposed to radon is substantially higher than the sum of risks due to smoking or radon in isolation (84). There is evidence that lung cancer may also be associated with wood smoke exposure (85, 86); wood is the primary source of heating in 14.4% of VT homes compared with only 1.7% nationally (53).

Lifestyle factors also play key roles in cancer incidence and many behavioral risk factors vary by rural/non rural status (87). Compared with the United States, NNE has a relatively good record for cancer screening, overweight/obesity, exercise, and nutrition, but somewhat worse for smoking history and alcohol use. The higher incidence of lung/bronchus, esophagus, and bladder cancer could be in part attributable to this region's higher proportion of people who have ever smoked, because past smoking influences cancer risk after a corresponding latency period; it has been reported previously that rural areas have higher incidence of tobacco-related cancers than urban areas (88), and associations have been identified in this area between county-level ever smoking and lung cancer mortality (77) in women. Although NNE has a higher prevalence of heavy drinking than the United States, this is not consistently reflected in all of the alcohol-related cancers (89); NNE has higher incidence than the United States of breast, esophageal, and oral cavity/pharyngeal cancers, but lower or comparable incidence of colorectal, liver, and laryngeal cancers. However, while the incidence of liver cancer in NNE is significantly lower than the United States, ME and NH are seeing increases in incidence that contrast with the national downward trend. Important causes of liver cancer include alcoholism and chronic infectious hepatitis (90, 91). In 2020, ME had the highest incidence by far of acute hepatitis C, almost twice that of the second ranked state (ME 11.9, Florida, 6.1 per 100,000 per year), and ME ranked second in the nation for incidence of acute hepatitis B (54, 55), so we would expect to see increases in liver cancer after an appropriate latency period. Both ME and VT have experienced substantial recent increases in a potentially related trend, drug overdose deaths (92).

Rural residents have previously been noted to have poorer access to health care, screening, and oncology services than their urban counterparts (93). There is also evidence that rural residents approach health and prevention differently than urban dwellers, with a fatalistic view that may reduce acceptance of preventive services (94, 95). However, it is not clear that poor access to health care accounts for the patterns we report here. Low incidence of cancers of the cervix and colorectum in NNE may be due to effective, long-standing screening programs that remove precancerous lesions to prevent future cancers. For breast cancer, the high incidence and low mortality that we observed relative to the United States may indicate success in breast cancer screening. However, reproductive factors also play a role (96) in breast cancer etiology, and higher incidence in NNE may also be related to lower fertility rates and more women having children at older ages (52). More parsimonious use of PSA screening in NNE may be associated with lower incidence of prostate cancer through reductions in overdiagnosis (97), although we saw significantly lower incidence in ME and VT than the United States and significantly higher in NH, a pattern that is hard to explain. Thyroid cancer incidence is higher in NNE than the United States, while late-stage diagnoses and mortality are comparable with the United States. This pattern is consistent with overdiagnosis which, in other studies, has been associated with higher health care utilization (98). The increasing incidence of thyroid cancers is thought to be due largely to incidental diagnosis during medical imaging and these tend to be primarily small, papillary cancers which have an excellent prognosis (99). In contrast, a trend toward less frequent use of hysterectomy may underlie the significant increase in uterine cancer being seen nationally (100, 101). In the Northeast, hysterectomy prevalence has historically been lower, and statistical adjustment for hysterectomy blunts the geographic differences in uterine cancer incidence (64, 102).

Interestingly, Zahnd analysis of cancer incidence in rural and urban residents highlighted some patterns that were not seen in NNE: (88) whereas throughout the United States, rural residents had higher incidence of colorectal and kidney cancer, our analyses showed lower incidence of these cancers in NNE; and whereas rural areas in the United States had lower incidence of thyroid cancer and non–Hodgkin lymphoma, we found these cancers were more common in NNE. In future analyses, it would be useful to compare incidence and mortality in rural and non-rural areas subdivisions of NNE, and to explore smaller areas to identify disparities that could potentially be addressed through educational outreach. However, it has been noted that even within small geographic areas, there are important differences in the attitudes of residents of different rural communities that mean educational interventions designed for one rural community may not be effective in another (103). Thus, the challenges are not only to identify cancer disparities in smaller geographic areas, but also to tailor interventions very specifically to different rural communities.

Limitations of these analyses include the small numbers that prevented detailed comparisons by cancer site for race/ethnic minority subgroups. We did not examine variability in cancer or cancer risk factor metrics by county or by sex, and these are future avenue of research to identify disparities. Although we pooled the three states for comparison with the rest of the country, our data indicate heterogeneity in various cancer and risk factor metrics that may obscure important trends at the state or county level and should be investigated further. However, recognizing these differences between the three states, we provide detailed state data in our supporting information.

## Conclusions

The tri-state area of ME, NH, and VT has higher cancer incidence and mortality than the United States, a pattern that contrasts with the Northeast region as a whole, which had lower cancer mortality overall than the United States despite significantly higher incidence. Within the tri-state area, variability was seen in these measures by state, race/ethnicity subgroup, and cancer site, and it seems likely that many factors underlie these patterns. In view of the known disadvantages experienced by rural populations, further exploration of these trends by county and by rural/non-rural residence is warranted. An understanding of individual risk factor data, not generally available in cancer registries, is needed to identify modifiable factors and ultimately improve population health in NNE.

## Supplementary Material

Supplementary Table S1 Data Quality criteria at 24 months, by state and diagnosis year, 2013-2017

Supplementary Table S2 Comparison of Age-standardized Incidence of Leading Cancer Sites, All Races, for Maine, New Hampshire, Vermont with United States 2013–2017

Supplementary Table S3 Patterns of incidence and mortality in Northern New England compared to the United States, among non-Hispanic White adults

Supplementary Table S4 Age-standardized Incidence of Cancer Maine, New Hampshire, Vermont, US Census Regions with the United States, All Races and by Race/Ethnicity, 2013-2017

Supplementary Table S5 Comparison of Age-standardized Cancer Mortality*, All Races, for Maine, New Hampshire, Vermont with the United States, 2013–2017

Supplementary Table S6 Northern New England state and population characteristics

Supplementary Figure 1 United States Regions used in the analyses; Age-adjusted Cancer Incidence in the United States, 2015-2019, all races combined; Age-adjusted Cancer Mortality in the United States, 2015-2019, all races combined

Supplementary Figure 2 Average Annual Percent Change (APC) in Age-Adjusted Cancer Incidence (2014-2018); Figure 2b Average Annual Percent Change (APC) in Age-Adjusted Cancer Mortality (2014-18)
