# Supplementary material for: Cancer Epidemiology in the Northeastern United States (2013–2017)
Source: Cancer Res Commun. 2023 Aug 14;3(8):1538–50. doi: 10.1158/2767-9764.CRC-23-0152 (PMC10424700; doi:10.1158/2767-9764.CRC-23-0152)
Supplement: Supplementary Table S1 — Data Quality criteria at 24 months, by state and diagnosis year, 2013-2017 [file crc-23-0152-s01.pdf]

Supporting Information Table S1 Data Quality criteria at 24 months, by state and diagnosis year, 2013-2017

	% Completeness Adjusted for Duplicates			% Death Clearance Only			All Invasive Age Adjusted Rate		
**Year**	**Maine**	**New Hampshire**	**Vermont**	**Maine**	**New Hampshire**	**Vermont**	**Maine**	**New Hampshire**	**Vermont**
2013	105.7	109.2	102.0	2.1	1.4	1.4	469.7 (459.4 - 480.2)	495.7 (484.7 - 506.9)	449.7 (434.6 - 465.3)
2014	111.1	111.9	106.9	2.1	1.0	1.8	480.4 (470.0 - 490.9)	480.1 (469.3 – 491.0)	466.4 (451.1 - 482.1)
2015	109.0	110.8	101.2	2.0	1.1	1.1	474.1 (463.8 - 484.5)	477.0 (466.3 - 487.8)	447.3 (432.4 - 462.6)
2016	110.4	109.1	104.3	2.1	1.4	1.8	475.7 (465.5 - 486.2)	486.7 (476.1 - 497.6)	453.5 (438.5 - 468.9)
2017	107.9	104.3	105.3	2.2	1.2	1.6	471.6 (461.5 - 481.9)	473.6 (463.2 - 484.2)	454.4 (439.6 - 469.7)

Data: https://www.naaccr.org/wp-content/uploads/2020/06/CINA.2013-2017.appendix.d.pdf (60)
