# Supplementary material for: Cancer Epidemiology in the Northeastern United States (2013–2017)
Source: Cancer Res Commun. 2023 Aug 14;3(8):1538–50. doi: 10.1158/2767-9764.CRC-23-0152 (PMC10424700; doi:10.1158/2767-9764.CRC-23-0152)
Supplement: Supplementary Table S2 — Comparison of Age-standardized Incidence of Leading Cancer Sites, All Races, for Maine, New Hampshire, Vermont with United States 2013–2017 [file crc-23-0152-s02.pdf]

Supporting Information Table S2 Comparison of Age-standardized Incidence*,¶ of Leading Cancer Sites, All Races, for Maine, New Hampshire, Vermont with United States 2013–2017

	All States		Northeast		Maine		New Hampshire		Vermont	(Population: 3,124,796)
**Leading Cancer Sites**	**Rate (95% CI)**	**Count**	**Rate (95% CI)**	**Count**	**Rate (95% CI)**	**Count**	**Rate (95% CI)**	**Count**	**Rate (95% CI)**	**Count**
All Invasive	440.6 (440.3, 440.9)	8,213,630	472.7# (472.0, 473.5)	1,623,337	464.3# (459.7, 468.9)	43,121	476.0# (471.2, 480.8)	41,014	446.9 (440.2, 453.7)	18,459
Brain and Other Nervous System	6.3 (6.3, 6.3)	110,298	6.7# (6.6, 6.8)	21,157	7.4# (6.8, 8.1)	600	7.4# (6.7, 8.0)	574	7.0 (6.1, 7.9)	253
Breast (Female)	125.2 (125.0, 125.5)	1,214,284	134.1# (133.6, 134.7)	239,958	126.8 (123.4, 130.3)	5,980	144.2# (140.6, 148.0)	6,390	130.9 (125.8, 136.1)	2,750
Cervix Uteri	7.6 (7.5, 7.6)	63,496	7.0# (6.9, 7.2)	10,927	5.9# (5.1, 6.8)	221	4.7# (4.0, 5.5)	174	4.3# (3.3, 5.5)	71
Colon and Rectum	37.8 (37.7, 37.9)	699,987	38.2# (38.0, 38.4)	131,346	36.0# (34.8, 37.3)	3,331	36.1 (34.8, 37.5)	3,061	33.8# (32.0, 35.7)	1,392
Corpus Uteri	26.0 (25.9, 26.1)	265,225	30.7# (30.4, 30.9)	58,485	30.6# (29.0, 32.2)	1,581	30.3# (28.7, 31.9)	1,438	33.5# (31.0, 36.0)	761
Esophagus	4.4 (4.4, 4.4)	84,881	4.8# (4.7, 4.9)	17,096	6.5# (6.0, 7.1)	638	6.5# (6.0, 7.1)	585	5.6# (4.9, 6.3)	246
Gallbladder	1.1 (1.1, 1.1)	20,076	1.2# (1.2, 1.3)	4,275	0.8# (0.6, 1.0)	75	1.2 (0.9, 1.4)	94	1.0 (0.7, 1.4)	43
Kidney and Renal Pelvis	16.6 (16.6, 16.7)	309,230	16.5 (16.3, 16.6)	56,312	16.3 (15.4, 17.2)	1,515	15.4 (14.6, 16.3)	1,320	14.4# (13.2, 15.7)	581
Larynx	3.2 (3.2, 3.2)	61,877	3.2 (3.1, 3.3)	11,455	3.6 (3.3, 4.0)	360	2.9 (2.5, 3.3)	258	3.2 (2.7, 3.9)	136
Leukemia	13.9 (13.8, 13.9)	249,547	14.9# (14.8, 15.0)	49,586	14.3 (13.5, 15.1)	1,273	13.5 (12.7, 14.4)	1,091	11.7# (10.7, 12.9)	462
Liver	6.8 (6.7, 6.8)	134,328	6.6# (6.5, 6.6)	23,924	4.1# (3.7, 4.5)	418	4.8# (4.4, 5.3)	444	4.4# (3.8, 5.1)	189
Lung and Bronchus	56.4 (56.3, 56.5)	1,071,089	58.6# (58.4, 58.9)	206,589	69.2# (67.5, 71.0)	6,700	61.9# (60.2, 63.6)	5,476	59.7# (57.3, 62.1)	2,557
Melanoma of the Skin	22.3 (22.2, 22.4)	404,686	21.7# (21.6, 21.9)	72,990	26.1# (25.0, 27.3)	2,291	32.1# (30.9, 33.4)	2,688	37.6# (35.6, 39.6)	1,503
Myeloma	6.7 (6.7, 6.8)	126,566	7.2# (7.1, 7.3)	25,102	5.8# (5.3, 6.4)	566	5.8# (5.3, 6.3)	507	5.3# (4.6, 6.0)	227
Non-Hodgkin Lymphoma	19.1 (19.0, 19.1)	349,576	20.9# (20.8, 21.1)	70,989	20.4# (19.5, 21.4)	1,871	20.7# (19.7, 21.7)	1,757	19.9 (18.5, 21.4)	811
Oral Cavity and Pharynx	11.7 (11.7, 11.8)	223,501	11.5# (11.4, 11.6)	40,272	13.2# (12.4, 14.0)	1,246	11.9 (11.1, 12.6)	1,060	11.8 (10.7, 12.9)	502
Ovary	10.7 (10.6, 10.8)	104,063	11.3# (11.1, 11.5)	20,532	9.2# (8.3, 10.2)	432	9.7 (8.8, 10.7)	447	9.9 (8.6, 11.5)	208
Pancreas	12.3 (12.2, 12.3)	232,485	13.4# (13.2,13.5)	47,120	12.2 (11.5, 12.9)	1,179	11.9 (11.2, 12.7)	1,047	11.3 (10.3, 12.4)	482
Prostate	103.0 (102.8, 103.2)	953,643	113.7# (113.1, 114.2)	191,792	86.7# (84.0, 89.4)	4,226	108.1# (105.0, 111.3)	4,921	85.5# (81.5, 89.6)	1,884
Stomach	6.4 (6.4, 6.4)	118,768	7.4# (7.3, 7.5)	25,455	5.3# (4.8, 5.8)	508	5.2# (4.7, 5.7)	455	5.6 (4.9, 6.4)	226
Thyroid	14.3 (14.2, 14.3)	240,311	19.2# (19.0, 19.4)	57,646	15.7# (14.8, 16.7)	1,187	15.5 (14.5, 16.4)	1,126	14.2 (12.9, 15.7)	484
Urinary Bladder (includes in situ)	19.8 (19.7, 19.8)	370,199	23.4# (23.2, 23.5)	82,059	26.5# (25.5, 27.6)	2,563	26.9# (25.8, 28.1)	2,322	23.1# (21.6, 24.7)	981

*Rates are per 100,000 and age-adjusted to the 2000 US Std Population (19 age groups - Census P25-1130) standard; Confidence intervals (Tiwari, R.C. [2006] method) are 95% for rates.

¶ Incidence data are compiled from cancer registries that meet the data quality criteria for all years 2013–2017 (covering >99% of the U.S. population).

# The rate ratio indicates that the rate is significantly different than the rate for All States after adjusting for multiple testing (p<0.01).

^ Statistics not displayed due to fewer than 16 cases to preserve confidentiality.

Data: NPCR and SEER Incidence - U.S. Cancer Statistics Public Use Database, 2019 submission (2001-2017) (25)

Software: Surveillance Research Program, National Cancer Institute SEER*Stat software (www.seer.cancer.gov/seerstat) version 8.3.9. (28)
