# Supplementary material for: Cancer Epidemiology in the Northeastern United States (2013–2017)
Source: Cancer Res Commun. 2023 Aug 14;3(8):1538–50. doi: 10.1158/2767-9764.CRC-23-0152 (PMC10424700; doi:10.1158/2767-9764.CRC-23-0152)
Supplement: Supplementary Table S3 — Patterns of incidence and mortality in Northern New England compared to the United States, among non-Hispanic White adults [file crc-23-0152-s03.pdf]

Supporting Information Table 3 Patterns of incidence and mortality* in Northern New England (NNE) compared to the United States, among non-Hispanic White adults

**Mortality, NNE v US**	**Incidence, NNE v US**		
	**Lower***	**Higher***	**Comparable**
**Lower**[1]	Cervix<br>Liver	Breast	
**Higher**[1]		Esophagus<br>Lung/bronchus<br>Urinary bladder<br>Uterus	Prostate
**Comparable**[1]	Colorectal<br>Kidney<br>Leukemia<br>Ovary		Brain<br>Gall bladder<br>Larynx<br>Leukemia<br>Myeloma<br>Non Hodgkin lymphoma<br>Oral cavity/pharynx<br>Pancreas<br>Stomach<br>Thyroid

*Bonferroni correction was used, statistical significance given by p<0.01.
