# Supplementary material for: Cancer Epidemiology in the Northeastern United States (2013–2017)
Source: Cancer Res Commun. 2023 Aug 14;3(8):1538–50. doi: 10.1158/2767-9764.CRC-23-0152 (PMC10424700; doi:10.1158/2767-9764.CRC-23-0152)
Supplement: Supplementary Table S4 — Age-standardized Incidence of Cancer Maine, New Hampshire, Vermont, US Census Regions with the United States, All Races and by Race/Ethnicity, 2013-2017 [file crc-23-0152-s04.pdf]

Supporting Information Table S4 Age-standardized Incidence* of Cancer Maine, New Hampshire, Vermont, US Census Regions with the United States, All Races** and by Race/Ethnicity, 2013-2017

	All Races		White		Black		American Indian/Alaska Native		Asian/Pacific Islander	
	Rate (95% CI)	Count	Rate (95% CI)	Count	Rate (95% CI)	Count	Rate (95% CI)	Count	Rate (95% CI)	Count
All States (Reference)	440.6 (440.3, 440.9)	8,213,630	443.0 (442.7, 443.3)	6,857,544	437.8 (436.9, 438.8)	899,653	281.8 (279.1, 284.4)	49,223	286.4 (285.3, 287.5)	269,604
Maine	464.3# (459.7, 468.9)	43,121	463.8# (459.2, 468.4)	42,222	324.0# (277.9, 375.0)	208	433.8# (373.0, 501.6)	222	235.9 (200.0, 276.3)	170
New Hampshire	476.0# (471.2, 480.8)	41,014	474.4# (469.5, 479.2)	39,718	310.9# (266.7, 359.8)	223	211.3 (151.5, 286.4)	51	256.9 (228.4, 287.8)	363
Vermont	446.9 (440.2, 453.7)	18,459	447.3 (440.6, 454.2)	18,111	304.9# (234.7, 387.9)	81	270.9 (185.8, 380.5)	41	265.7 (209.1, 331.7)	95
Northeast	472.7# (472.0, 473.5)	1,623,337	477.5# (476.7, 478.3)	1,371,207	440.3 (438.1, 442.5)	163,509	129.7# (124.1, 135.5)	2,229	305.9# (303.2, 308.6)	54,098
Midwest	452.5# (451.8, 453.2)	1,748,418	451.4# (450.6, 452.1)	1,542,646	459.3# (457.0, 461.7)	154,573	359.7# (351.3, 368.3)	8,105	259.6# (256.1, 263.2)	22,392
South	441.6# (441.1, 442.1)	3,091,595	440.9# (440.4, 441.5)	2,474,445	439.0 (437.7, 440.3)	503,545	305.8# (300.9, 310.7)	16,813	249.6# (247.2, 251.9)	48,128
West	402.3# (401.7, 403.0)	1,612,736	410.7# (410.0, 411.4)	1,349,005	392.0# (389.0, 395.0)	70,141	274.2# (270.3, 278.2)	20,892	299.5# (297.9, 301.1)	140,076

	Non-Hispanic All Races		Non-Hispanic White		Non-Hispanic Black		Non-Hispanic American Indian/Alaska		Non-Hispanic Asian/Pacific Islander	
	Rate (95% CI)	Count	Rate (95% CI)	Count	Rate (95% CI)	Count	Rate (95% CI)	Count	Rate (95% CI)	Count
All States (Reference)	453.0 (452.7, 453.4)	7,563,206	457.6 (457.3, 458.0)	6,264,090	447.3 (446.4, 448.3)	880,509	380.4 (376.8, 384.0)	46,737	289.7 (288.5, 290.8)	265,014
Maine	465.9# (461.3, 470.5)	42,945	465.3# (460.6, 470.0)	42,074	336.0# (287.8, 389.6)	204	448.1 (385.2, 518.1)	220	234.5# (198.0, 275.6)	164
New Hampshire	478.4# (473.6, 483.3)	40,590	477.0# (472.1, 482.0)	39,379	332.9# (283.9, 387.2)	208	238.5# (169.5, 326.6)	49	257.1 (228.3, 288.2)	357
Vermont	449.0 (442.3, 455.9)	18,381	449.4 (442.5, 456.3)	18,042	327.2# (251.0, 417.3)	80	288.4 (197.9, 405.8)	41	269.5# (212.2, 336.3)	95
Northeast	482.1# (481.3, 482.9)	1,514,178	488.0# (487.1, 488.9)	1,284,607	460.3# (458.0, 462.7)	151,746	258.0# (246.6, 269.7)	2,096	308.8# (306.1, 311.5)	53,276
Midwest	457.6# (456.9, 458.3)	1,704,331	456.8 (456.1, 457.6)	1,502,413	464.5# (462.1, 466.8)	153,840	422.9# (412.9, 433.0)	7,826	260.8# (257.2, 264.4)	22,063
South	454.8# (454.3, 455.4)	2,843,213	458.2 (457.6, 458.8)	2,244,851	444.5# (443.2, 445.8)	498,322	380.8 (374.7, 386.9)	16,294	248.8# (246.5, 251.2)	46,696
West	419.0# (418.3, 419.7)	1,373,941	429.8# (428.9, 430.6)	1,121,212	413.1# (409.9, 416.3)	68,810	383.5 (377.9, 389.2)	19,390	305.0# (303.4, 306.6)	138,137

	Hispanic All Races		Hispanic White		Hispanic Black		Hispanic American Indian/Alaska Native		Hispanic Asian/Pacific Islander	
	Rate (95% CI)	Count	Rate (95% CI)	Count	Rate (95% CI)	Count	Rate (95% CI)	Count	Rate (95% CI)	Count
All States (Reference)	338.4 (337.5, 339.3)	649,935	337.7 (336.8, 338.6)	593,223	227.4 (223.9, 230.9)	19,123	47.4 (45.3, 49.6)	2,484	186.2 (180.2, 192.3)	4,586
Maine	266.1# (226.2, 310.5)	176	255.5# (213.9, 302.3)	148	^ (^ , ^)	^	^ (^ , ^)	^	^ (^ , ^)	^
New Hampshire	310.1 (278.2, 344.3)	424	282.5# (250.3, 317.4)	339	^ (^ , ^)	^	^ (^ , ^)	^	^ (^ , ^)	^
Vermont	216.0# (166.6, 274.2)	78	218.2# (165.5, 280.9)	69	^ (^ , ^)	^	^ (^ , ^)	^	^ (^ , ^)	^
Northeast	374.8# (372.5, 377.2)	108,670	369.2# (366.6, 371.8)	86,369	284.2# (278.8, 289.7)	11,742	14.1# (11.6, 17.0)	131	194.1 (180.1, 208.8)	818
Midwest	324.9# (321.5, 328.2)	44,087	323.0# (319.5, 326.5)	40,233	144.6# (133.0, 156.8)	733	69.8# (60.2, 80.3)	279	205.9 (181.6, 232.3)	329
South	337.2 (335.8, 338.6)	248,382	330.9# (329.5, 332.3)	229,594	211.0# (204.7, 217.3)	5,223	45.0 (40.6, 49.8)	519	301.3# (283.6, 319.7)	1,432
West	329.1# (327.7, 330.5)	238,795	338.0# (336.5, 339.5)	227,793	106.9# (100.4, 113.6)	1,331	57.4# (54.1, 60.8)	1,502	145.1# (138.0, 152.4)	1,939

*Rates are per 100,000 and age-adjusted to the 2000 US Std Population (19 age groups - Census P25-1130) standard; Confidence intervals (Tiwari, R.C. [2006] method) are 95% for rates.

**All race case counts include 39,346 (0.5%) other unspecified and 98,260 (1.2%) unknown.

^ Statistics not displayed due to fewer than 16 cases to preserve confidentiality.

The age-standardized rate is significantly different to the rate for Northeast, (p<0.01, overall significance level).

Note: The Race Recode for USCS variable contains “other unspecified” and “unknown” categories; these groups are coded as “unknown race” for analyses (30)

Data: (25). Population data are not available for “other race” and “unknown race” categories.
