# Supplementary material for: Cancer Epidemiology in the Northeastern United States (2013–2017)
Source: Cancer Res Commun. 2023 Aug 14;3(8):1538–50. doi: 10.1158/2767-9764.CRC-23-0152 (PMC10424700; doi:10.1158/2767-9764.CRC-23-0152)
Supplement: Supplementary Table S5 — Comparison of Age-standardized Cancer Mortality*, All Races, for Maine, New Hampshire, Vermont with the United States, 2013–2017 [file crc-23-0152-s05.pdf]

Supporting Information Table S5 Comparison of Age-standardized Cancer Mortality* , All Races, for Maine, New Hampshire, Vermont with the United States, 2013–2017

	**All States (Population: 1,603,405,284)**		**Northeast (Population: 280,102,739)**		**Maine (Population: 6,653,873)**		**New Hampshire (Population: 6,688,065)**		**Vermont (Population: 3,124,796)**	
**Leading Cancer Sites**	**Rate (95% CI)**	**Count**	**Rate (95% CI)**	**Count**	**Rate (95% CI)**	**Count**	**Rate (95% CI)**	**Count**	**Rate (95% CI)**	**Count**
All Invasive	158.3 (158.2, 158.5)	2,969,607	154.7# (154.3, 155.1)	546,567	172.0# (169.3, 174.7)	16,500	159.1 (156.4, 161.9)	13,690	164.7# (160.7, 168.7)	6,886
Brain and Other Nervous System	4.4 (4.4, 4.4)	81,246	4.3# (4.2, 4.3)	14,399	5.1# (4.7, 5.6)	465	4.8 (4.3, 5.3)	401	5.8# (5.0, 6.6)	228
Breast (Female)	20.3 (20.2, 20.4)	207,081	19.7# (19.5, 19.9)	38,405	18.6# (17.4, 19.9)	953	18.9 (17.6, 20.2)	880	17.7# (15.9, 19.6)	396
Cervix Uteri	2.3 (2.2, 2.3)	20,902	1.9# (1.8, 2.0)	3,343	1.6# (1.2, 2.1)	72	1.3# (0.9, 1.7)	56	1.1# (0.7, 1.7)	21
Colon and Rectum	13.9 (13.9, 14.0)	260,693	13.4# (13.3, 13.5)	47,385	12.8# (12.1, 13.6)	1,215	12.6# (11.9, 13.4)	1,078	14.8 (13.7, 16.1)	611
Corpus Uteri	2.3 (2.2, 2.3)	23,785	2.6# (2.5, 2.6)	5,105	2.7 (2.3, 3.2)	146	3.0# (2.5, 3.6)	139	3.6# (2.9, 4.6)	82
Esophagus	3.9 (3.9, 4.0)	75,615	4.1# (4.1, 4.2)	14,734	5.6# (5.1, 6.1)	552	5.4# (4.9, 5.9)	481	4.9# (4.2, 5.6)	214
Gallbladder	0.6 (0.6, 0.6)	10,925	0.6# (0.6, 0.7)	2,222	0.4 (0.3, 0.6)	40	0.7 (0.5, 0.9)	60	0.6 (0.4, 0.9)	24
Kidney and Renal Pelvis	3.7 (3.7, 3.7)	70,072	3.2# (3.2, 3.3)	11,412	4.1 (3.7, 4.6)	403	3.6 (3.2, 4.0)	316	3.6 (3.0, 4.2)	151
Larynx	1.0 (1.0, 1.0)	18,823	1.0 (0.9, 1.0)	3,435	1.3# (1.1, 1.5)	127	0.8 (0.6, 1.0)	66	0.8 (0.6, 1.2)	35
Leukemia	6.4 (6.4, 6.5)	116,774	6.3# (6.2, 6.4)	21,685	6.4 (5.8, 6.9)	596	6.2 (5.6, 6.7)	512	6.4 (5.6, 7.2)	259
Liver	4.9 (4.9, 5.0)	96,772	4.2# (4.2, 4.3)	15,459	3.3# (2.9, 3.7)	327	3.6# (3.2, 4.1)	330	3.0# (2.5, 3.6)	133
Lung and Bronchus	40.2 (40.1, 40.3)	760,138	38.2# (38.0, 38.4)	135,015	48.8# (47.4, 50.2)	4,753	42.5# (41.1, 43.9)	3,691	42.8 (40.8, 44.9)	1,822
Melanoma of the Skin	2.4 (2.4, 2.4)	43,847	2.2# (2.2, 2.3)	7,718	2.7 (2.3, 3.1)	248	2.8 (2.4, 3.2)	229	2.5 (2.0, 3.0)	103
Myeloma	3.3 (3.2, 3.3)	60,732	3.1# (3.1, 3.2)	10,982	3.2 (2.8, 3.6)	308	3.0 (2.6, 3.4)	250	3.2 (2.7, 3.9)	136
Non-Hodgkin Lymphoma	5.5 (5.5, 5.5)	101,381	5.5 (5.4, 5.6)	19,267	6.1 (5.6, 6.6)	568	5.6 (5.1, 6.1)	464	5.9 (5.2, 6.8)	237
Oral Cavity and Pharynx	2.5 (2.5, 2.5)	48,304	2.2# (2.1, 2.2)	7,842	2.7 (2.4, 3.1)	269	2.5 (2.1, 2.8)	223	3.0 (2.5, 3.6)	130
Ovary	6.9 (6.8, 6.9)	70,807	7.0 (6.8, 7.1)	13,659	6.6 (5.9, 7.3)	341	7.2 (6.4, 8.0)	330	6.8 (5.7, 8.0)	150
Pancreas	11.0 (10.9, 11.0)	207,797	11.3# (11.2, 11.4)	40,316	11.1 (10.4, 11.8)	1,084	10.4 (9.7, 11.1)	911	10.7 (9.8, 11.8)	458
Prostate	19.1 (19.0, 19.2)	145,728	18.3# (18.1, 18.5)	26,235	20.4 (19.0, 21.9)	807	18.9 (17.4, 20.4)	661	19.1 (17.0, 21.3)	326
Stomach	3.1 (3.0, 3.1)	56,493	3.1 (3.1, 3.2)	10,937	2.0# (1.7, 2.3)	191	2.0# (1.7, 2.3)	172	2.4 (1.9, 2.9)	100
Thyroid	0.5 (0.5, 0.5)	9,510	0.5 (0.5, 0.5)	1,782	0.4 (0.3, 0.5)	37	0.4 (0.3, 0.5)	31	0.6 (0.4, 0.9)	27
Urinary Bladder (includes in situ)	4.4 (4.3, 4.4)	81,089	4.6# (4.5, 4.7)	16,567	5.4# (5.0, 5.9)	522	5.2# (4.7, 5.7)	441	5.6# (4.8, 6.3)	226

*Rates are per 100,000 and age-adjusted to the 2000 US Std Population (19 age groups - Census P25-1130) standard; Confidence intervals (Tiwari method) (63)are 95% for rates and ratios.

The rate ratio indicates that the rate is significantly different than the rate for All States after adjusting for multiple testing (p<0.01).

Data: Mortality -Surveillance, Epidemiology, and End Results (SEER) Program SEER*Stat Database: Mortality - All COD, Aggregated With State, Total U.S. (1990-2017) (26) Underlying mortality data provided by NCHS (www.cdc.gov/nchs).

Software: Surveillance Research Program, National Cancer Institute SEER*Stat software (www.seer.cancer.gov/seerstat) version 8.3.9. (28)
