# Supplementary material for: Cancer Epidemiology in the Northeastern United States (2013–2017)
Source: Cancer Res Commun. 2023 Aug 14;3(8):1538–50. doi: 10.1158/2767-9764.CRC-23-0152 (PMC10424700; doi:10.1158/2767-9764.CRC-23-0152)
Supplement: Supplementary Table S6 — Northern New England state and population characteristics [file crc-23-0152-s06.pdf]

Supporting Information Table S6 State and population characteristics

**Geographic**	**United States**	**Maine**	**New Hampshire**	**Vermont**
Population, 2020 (37)	331,449,281	1,362,359	1,377,529	643,077
Land [square miles] 2010 (20)	3,531,905	30,843	8,953	9,217
Population density per square mile 2020 [rank] (37)	93.8 [NA]	44.2 [40]	153.8 [23]	69.8 [33]
Population growth 1970-2020 (37)	63.1%	37.3%	86.7%	44.8%
Latitude [range North-South] (24)	N71°30′-N18°52′	N47°28′-N42°58′	N45°21′–N42°42′	N45°00′–N42°43′
Average winter temperature 1971-2000 [rank] (22)	Range 2.6 - 67.4° F	16.8° F [47]	21.1° F [43]	19.4° F [45]
Population living in rural areas 2018 - [range] [rank] (23)	25.4% [0.1-87.2%]	52.5% [8]	39.6% [14]	71.2% [3]
**State Infrastructure**	**United States**	**Maine**	**New Hampshire**	**Vermont**
Excise Tax on Packs of Cigarettes as of 12/31/2021 (50)	Median $1.34	$2.00	$1.78	$3.08
State Liquor Excise Tax per Gallon 2021 (50)	Median $5.50	$5.79	$0.00	$6.78
State Tax Collections per Capita 2020 (44)	$3,217	$3,561	$2,075	$5,318
Superfund National Priorities List (NPL) sites				
Current NPL sites as of Oct. 2022 [50 states+DC] (35)	1,312	11	20	12
Proposed NPL sites as of Sep. 2022 [50 states+DC] (36)	39	0	1	0
Deleted NPL sites as of Sep. 2022 [50 states+DC] (34)	442	5	1	2
Total (current, proposed & deleted) NPL sites [Sep./Oct. 2022]	1,793	16	22	14
Total NPL sites per million population [Sep./Oct. 2022]	Median 5.8, Range 0.6-26.3	11.7	16.0	21.8
Total NPL sites per 10,000 square miles land [Sep./Oct. 2022]	Median 6.2, Range 0.2-206.7	5.2	24.6	15.2
**Demographics**	**United States**	**Maine**	**New Hampshire**	**Vermont**
White alone race 2020 (21)	62%	91%	88%	90%
Black or African American alone race 2020 (21)	12%	2%	1%	1%
American Indian and Alaska native alone race 2020 (21)	6%	1%	3%	2%
Asian only race 2020 (21)	1%	1%	0%	0%
2 or more races 2020 (21)	10%	5%	6%	6%
Hispanic or Latino 2020 (39)	19%	2%	4%	2%
Speak English only or speak English "very well", age 18+ 2020 (38)	95.1%	99.0%	98.5%	99.2%
Born in state of residence 2020 (41)	58.2%	62.2%	41.0%	49.5%
Born outside United States 2020 (41)	13.5%	3.6%	6.1%	4.6%
Lived in different state 1 year ago 2020 (41)	2.3%	2.6%	3.9%	3.8%
Age, median 2020 (40)	38.2	44.8	43.0	42.8
Age >65 years 2020 (40)	16.0%	20.6%	18.1%	19.4%
Disabled [civilian noninstitutionalized population] 2020 (41)	12.7%	15.9%	12.8%	14.2%
Civilian Veteran 2020 (41)	7.1%	9.3%	8.5%	6.9%
Education: Did not graduate high school, aged 25+ 2020 (41)	11.5%	6.8%	6.7%	6.5%
Education: High school graduate or higher, aged 25+ 2020 (41)	88.5%	93.2%	93.3%	93.5%
Education: Bachelor's degree or higher, aged 25+, 2020 (41)	32.9%	32.5%	37.6%	39.7%
Median household income 2020 (42)	$64,994	$59,489	$77,923	$63,477
People below poverty level 2020 (43)	12.8%	11.1%	7.4%	10.8%
Registered Democrat / Republicans / Independent 2014 (45)		47% / 36% / 17%	44% / 35% / 20%	57% / 29% / 14%
**Homes**	**United States**	**Maine**	**New Hampshire**	**Vermont**
Owner-occupied homes 2020 (53)	64.4%	72.9%	71.2%	71.3%
Home has high radon level, 2008-17 [>=4 ng/mL] (5)	7%	37%	35%	22%
Home has a computer 2020 (41)	91.9%	91.1%	93.7%	91.3%

Home has broadband access 2020 (41)	85.2%	84.3%	88.8%	83.0%
Home heated primarily by wood 2021 (53)	1.7%	9.6%	6.6%	14.4%
**Health care**	**United States**	**Maine**	**New Hampshire**	**Vermont**
Date State implemented Medicaid expansion (46)		01/10/19 [retroactive to	8/15/14	1/1/14
Registered nurses per 100,000 population 2015-19 (57)	Median 1,055, Range 758-1639	1,129	1,152	1,097
Physicians per 100,000 population 2019 (57)	Median 320, Range 210-931	374	382	518
Current lack of health insurance [18-64y] 2020 [95% CI]* (47)	13.0% [11.6-14.6]	12.0% [10.7-13.5]	9.8% [8.4%-11.3]	8.6% [7.4-10.1]
**Health**	**United States**	**Maine**	**New Hampshire**	**Vermont**
Cholesterol checked in past 5 years [>=18y] 2019 [95% CI]* (47)	88.9% [87.9-90.0]	88.0% [86.4-89.5]	88.7% [86.8-90.4]	84.8% [82.7-86.6]
Diabetes [>= 18y] 2020 [95% CI]* (47)	9.4% [9.0-9.9]	8.3% [7.6-9.0]	7.5% [6.7-8.2]	6.8% [6.1-7.6]
Chronic obstructive pulmonary disease [>= 18y] 2020 [95% CI]* (47)	5.6% [5.2-6.2]	6.9% [6.3-7.6]	5.7% [5.0-6.5]	5.2% [4.6-5.9]
Chronic kidney disease [>= 18y] 2020 [95% CI]* (47)	2.6% [2.5-2.8]	2.6% [2.2-3.0]	1.6% [1.3-2.0]	1.8% [1.5-2.2]
Fertility rate [# births per 1,000 women aged 15-44] [2016-21] [rank] (33)	56.3	49.9 [45]	49.9 [44]	44.9 [50]
Births to women aged 30 or older as % of all births [2016-21] (33)	50.3%	58.8%	59.2%	52.8%
Hysterectomy prevalence [Non Hispanic whites >=20y] 2004-08 [±SE]* (63)	19.9% ±0.08	18.1% ±0.42	15.4% ±0.34	14.0% ±0.30
**Cancer screening and prevention**	**United States**	**Maine**	**New Hampshire**	**Vermont**
Cancer survivors: crude 5-year limited duration cancer prevalence [%] [rank] (56)	1.77%	2.24% [1]	2.22% [2]	2.16% [4]
Met USPSTF colorectal cancer screening guidelines [50-75y] 2020 [95% CI] (51)	Median 74.3%	81.2% [79.6-82.7]	77.8% [75.9-79.7]	77.5% [75.6-79.5]
Never screened for colorectal cancer [50-75y] 2020 [95% CI] (51)	Median 18.2%	13.4% [12.0-14.8]	15.0% [13.4-16.7]	15.1% [13.4-16.9]
Mammogram in past 2 years [women 50-74y] 2020 [95% CI]* (47)	77.9% [76.2-78.7]	82.2% [80.0-84.1]	77.0% [74.3-79.6]	73.9% [70.9-76.7]
Had Pap test in past 3 years [women 21-65y] 2020 [95% CI]* (47)	78.0% [77.1-78.7]	80.3% [77.8-82.6]	79.2% [76.2-81.9]	75.4% [72.3-78.4]
Lung cancer screening among those eligible, 2021 [rank] (5)	6%	12% [5]	11% [6]	13% [3]
Had PSA Test in past 2 years [men >=40y] 2020 [95% CI] (51)	Median 31.8%	22.5% [20.2-24.8]	25.4% [23.0-27.7]	20.0% [17.9-22.0]
Up-to-date with HPV vaccinations [13-17y] 2020 [95% CI] (51)	58.6% [57.3-60.0]	63.5% [57.5-69.1]	68.8% [63.1-73.9]	70.5% [64.5-75.8]
Acute Hepatitis B infections [per 100,000] 2019 [rank] (54)	1.0	4.3 [1]	0.4 [35]	1.4 [12]
Acute Hepatitis C infections [per 100,000] 20 [rank] (55)	1.5	11.9 [1]	0.4 [30]	0.2 [36]
**Behavioral risk factors**	**United States**	**Maine**	**New Hampshire**	**Vermont**
Does muscle strengthening exercises >=2/week 2019 [95% CI] (51)	Median 35.7%	30.6% [29.1-32.1]	37.2% [35.3-39.1]	39.7% [37.7-41.6]
Consumed vegetables >=1/day 2019 [95% CI] (51)	Median 79.7%	87.1% [85.7 - 88.5]	83.5% [81.8 - 85.3]	87.5% [85.9 - 89.0]
Consumed fruit >=1/day 2019 [95% CI] (51)	Median 60.7%	63.9% [62.0 - 65.8]	63.1% [60.8 - 65.3]	67.3% [65.1 - 69.5]
Current smokers [>=18y] 2020 [95% CI]* (47)	16.1% [14.2-17.5]	18.0% [16.7-19.5]	14.7% [13.3-16.3]	14.4% [12.9-15.9]
Quit attempts in past year [current smokers >=18y] 2020 [95% CI]* (47)	54.7% [53.1-56.2]	48.5% [44.1-52.9]	54.7% [49.3-59.9]	52.6% [47.2-57.9]
Former smoker 2020 [95% CI] (51)	Median 25.2%	27.2% [25.8 - 28.6]	26.8% [25.1-28.5]	26.9% [25.2-28.6]
Never smoked 2020 [95% CI] (51)	Median 59.5%	54.9% [53.1-56.6]	58.7% [56.7-60.7]	59.1% [57.0-61.1]
Heavy drinking [>=18y] 2020 [95% CI]* (47)	6.9% [6.7-7.4]	8.5% [7.5 – 9.7]	8.4% [7.3 – 9.5]	10.2% [9.0 – 11.6]
Overweight [BMI 25.0-29.9] 2020 [95% CI]* (51)	Median 35.2%	33.0% [31.3-34.6]	35.1% [33.0 - 37.1]	35.3% [33.1-37.5]
Obesity [BMI 30.0-] 2020 [95% CI]* (51)	Median 31.9%	30.9% [29.2 - 32.6]	29.7% [27.8 - 31.6]	25.8% [24.0 - 27.6]
Reports good or better health 2020 [95% CI]* (51)	Median 86.7%	88.9% [87.9-89.9]	89.3% [88.1-90.4]	89.5% [88.3-90.7]

*Age-adjusted prevalence standardized to the age distribution of a specific population, usually the U.S > 2000 population

Publicly available data sources referenced to the main manuscript in () parentheses
