# Supplementary material for: Cancer Epidemiology in the Northeastern United States (2013–2017)
Source: Cancer Res Commun. 2023 Aug 14;3(8):1538–50. doi: 10.1158/2767-9764.CRC-23-0152 (PMC10424700; doi:10.1158/2767-9764.CRC-23-0152)
Supplement: Supplementary Figure 1 — United States Regions used in the analyses; Age-adjusted Cancer Incidence in the United States, 2015-2019, all races combined; Age-adjusted Cancer Mortality in the United States, 2015-2019, all races combined [file crc-23-0152-s07.pdf]

Supporting Information Figure 1a United States Regions used in the analyses

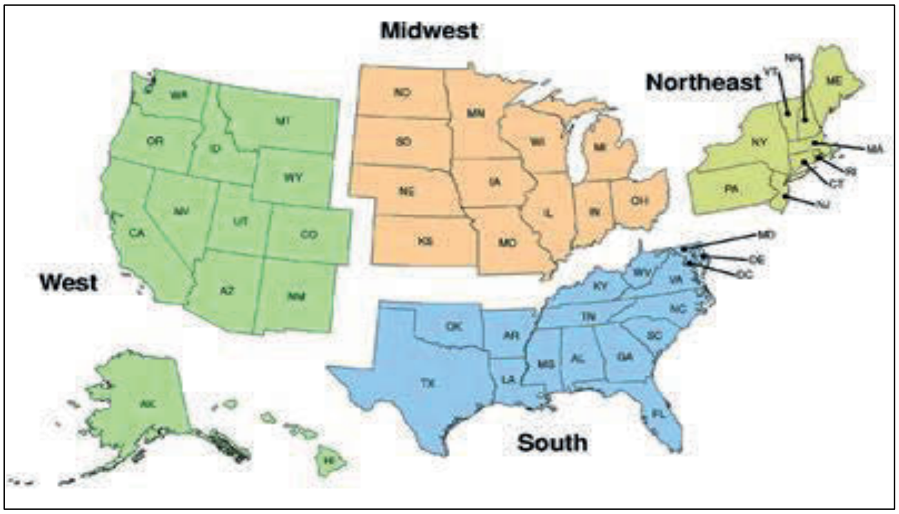

Supporting Information Figure 1b Age-adjusted Cancer Incidence in the United States, 2015-2019, all races combined

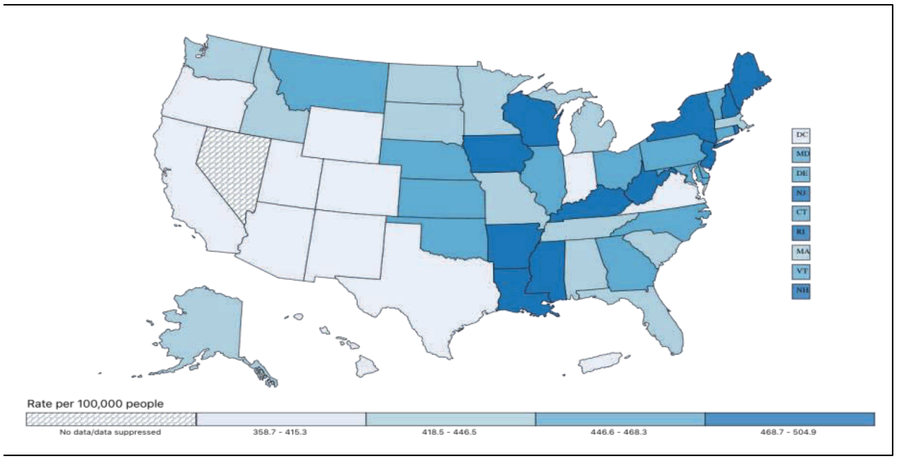

Rates are the number of cases (or deaths) per 100,000 people and are age-adjusted to the 2000 U.S. standard population
Data Figure 1a: https://www.cdc.gov/surveillance/nrevss/coronavirus/region.html (58)

Supporting Information Figure 1c Age-adjusted Cancer Mortality in the United States, 2015-2019, all races combined

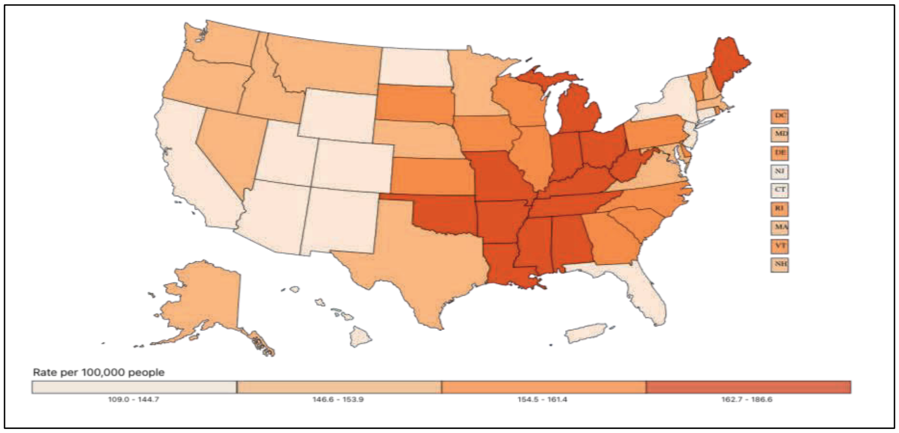

Rates are the number of cases (or deaths) per 100,000 people and are age-adjusted to the 2000 U.S. standard population
Data Fig 1b, 1c: www.cdc.gov/cancer/dataviz, released in November 2022.(56)
