# Supplementary material for: Cancer Epidemiology in the Northeastern United States (2013–2017)
Source: Cancer Res Commun. 2023 Aug 14;3(8):1538–50. doi: 10.1158/2767-9764.CRC-23-0152 (PMC10424700; doi:10.1158/2767-9764.CRC-23-0152)
Supplement: Supplementary Figure 2 — Average Annual Percent Change (APC) in Age-Adjusted Cancer Incidence (2014-2018); Figure 2b Average Annual Percent Change (APC) in Age-Adjusted Cancer Mortality (2014-18) [file crc-23-0152-s08.pdf]

Supporting Information Figure S2a Average Annual Percent Change (APC) in Age-Adjusted Cancer Incidence (2014-2018)

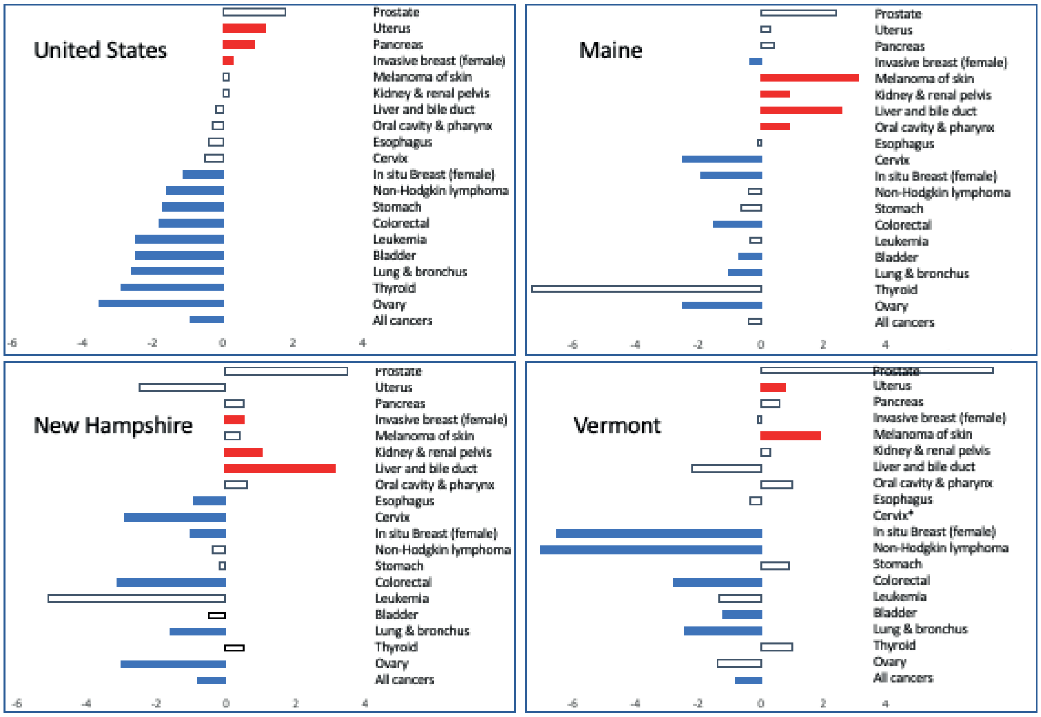

Supporting Information Figure S2b Average Annual Percent Change (APC) in Age-Adjusted Cancer Mortality (2014-18)

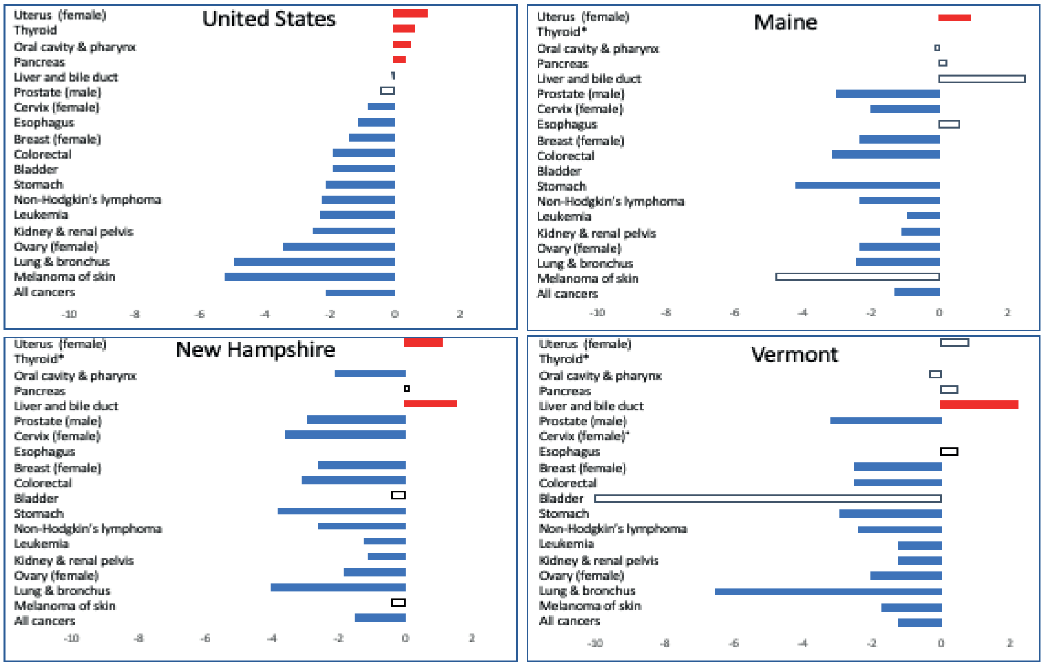

Data: State Cancer Profiles, National Institutes of Health, National Cancer Institute: www.statecancerprofiles.gov (32)

Horizontal axis: Annual percent change (APC) in (1A) incidence or (1B) mortality for all races combined.

Red bars: Statistically significant increase (APC>0%) in incidence (1A) or mortality (1B) over time.

Blue bars: Statistically significant decrease (APC<0%) in incidence (1A) or mortality (1B) over time.

White bars: No significant APC.

*Data suppressed due to small numbers
